# NiF_2_ Microspheres Grown on Nickel Foam for Efficient Hydrogen Evolution in Both Acidic and Alkaline Media

**DOI:** 10.3390/ma19183871

**Published:** 2026-09-11

**Authors:** Jun Yang, Zenghui Wan, Long Yu, Changlin Yu

**Affiliations:** 1 School of Chemical Engineering, Guangdong University of Petrochemical Technology, Maoming 525000, China; 2 Guangdong Provincial Key Laboratory of Green and Intelligent Agricultural Production, School of Environmental Science and Engineering, Guangdong University of Petrochemical Technology, Maoming 525000, China; 17755457610@163.com (Z.W.); longyu@gdupt.edu.cn (L.Y.)

**Keywords:** NiF_2_/NF, electrocatalysts, electrolytes, hydrogen evolution reaction

## Abstract

It is of technological significance to construct efficient electrocatalysts for hydrogen production. Here, a novel composite based on NiF_2_ microspheres in situ grown on nickel foam (NiF_2_/NF) has been synthesized by a facile solvothermal procedure. Benefiting from the synergistic contribution of NiF_2_ microspheres and NF substrate, the derived NiF_2_/NF composite demonstrated excellent electrocatalytic performance for the hydrogen evolution reaction (HER) in both acidic and alkaline media. Specifically, it can deliver current densities of 10, 100, and 300 mA cm^−2^ at low overpotentials of only 40, 134, and 195 mV in 0.5 M H_2_SO_4_ and 69, 132, and 175 mV in 1.0 M KOH. Furthermore, an alkaline electrolyzer assembled with NiF_2_/NF as both the cathode and anode only required a low cell voltage of 1.57 V to drive the current density of 10 mA cm^−2^ for overall water splitting. This work proposed a new way for developing wide-pH HER electrocatalysts with high performance.

## 1. Introduction

With the proposal of carbon neutrality and emission peak, hydrogen energy is being rapidly developed for its large energy density and environmental friendliness [1,2,3,4,5]. Water electrolysis is believed to be an effective technology for producing clean hydrogen with high purity and has been attracting considerable academic interest in recent years [6,7,8,9]. To date, platinum-based electrocatalysts are commonly accepted as the benchmark for the hydrogen evolution reaction (HER), but still limited by high costs and low reserves, as well as their poor electrochemical performance in non-acidic electrolytes [10,11,12,13]. It is therefore of economic and technological significance to explore cost-effective HER electrocatalysts with high activity and long stability, particularly ones that can work well in both acidic and alkaline media.

Nickel-based compounds, such as metal sulfides [14,15,16], selenides [17,18], phosphides [19,20], and nitrides [21,22,23], especially those directly grown on the conductive substrate, have shown great promise for efficiently splitting water to produce hydrogen, primarily due to their high abundance, good conductivity and low cost [24,25,26]. With the construction of self-supporting electrodes, not only can the charge transfer and electrolyte penetration be facilitated, but exfoliation of the electroactive component can be effectively avoided, thus leading to enhanced electrocatalytic activity and durability [26,27,28]. It has been reported that Ni_12_P_5_-Ni_2_P polymorph catalysts on coarsened nickel foam support, prepared through electrodeposition, hydrothermal reaction and subsequent phosphorization treatment, only required low overpotentials of 83 and 129 mV to drive 10 mA cm^−2^ for the HER in acidic and alkaline electrolytes, respectively [28].

Recently, accelerated efforts have also been devoted to the exploration of high-performance metal fluorides for energy storage and conversion [29]. For example, the self-supporting NiF_3_/Ni_2_P@CC electrode has been synthesized by successive hydrothermal, fluorination and phosphorization procedures and delivered a current density of 10 mA cm^−2^ at the overpotential of 121 mV for the HER [30]. Mu and co-workers prepared the reconstructed NiF_2_ by hydrothermal process, high-temperature treatment and electrochemical activation, which showed a HER overpotential of 101 mV at 10 mA cm^−2^ in 1.0 M KOH [31]. In another study, NiF_2_@Ni also exhibited moderate activity (172 mV@10 mA cm^−2^) only for alkaline HER [32]. Although such Ni-based electrode materials demonstrated encouraging prospects for the HER, the complex fabrication process inevitably causes high energy consumption and is unsuitable for mass production in industry. More importantly, further improvement in electrocatalytic HER properties, especially in terms of the wide pH range and the large current density, is sorely needed for industrial hydrogen generation.

In this work, an efficient self-supporting electrode, NiF_2_ microspheres directly grown on nickel foam (NiF_2_/NF), was fabricated successfully by a one-step solvothermal treatment, which is suitable for large-scale practical production. The as-prepared NiF_2_/NF electrocatalyst exhibited superior performance for the HER, with very low overpotentials of 40, 134, and 195 mV at 10, 100, and 300 mA cm^−2^ in acidic media. Even at a large current density of 500 mA cm^−2^, its overpotential was still as low as 226 mV. Under alkaline conditions, NiF_2_/NF also demonstrated excellent HER activity with low overpotentials of only 69, 132, and 175 mV to deliver current densities of 10, 100, and 300 mA cm^−2^, respectively. Moreover, NiF_2_/NF was found to be active towards overall water splitting, with a low cell voltage of 1.57 V to achieve a current density of 10 mA cm^−2^ in an alkaline electrolyzer.

## 2. Materials and Methods

### 2.1. Synthesis of NiF_2_/NF

The NiF_2_/NF was synthesized by a modified solvothermal method [33]. In a typical procedure, the nickel foam (NF) substrate (2 cm × 3 cm) was first cleaned with acetone, deionized water and absolute ethanol by ultrasonic treatment. A total of 300 mg of urea (Shanghai Aladdin Biochemical Technology Co., Ltd., Shanghai, China) was dissolved in 15 mL of absolute ethanol to form a clear solution; then, 3 mL of hydrofluoric acid (HF, 40%; Shanghai Macklin Biochemical Co., Ltd., Shanghai, China) was added under vigorous- stirring. The mixed solution and NF were subsequently transferred into a 25 mL Teflon-lined autoclave and heated at 120 °C for 12 h. After cooling down to room temperature, the sample was washed with deionized water and absolute ethanol several times, and then dried under vacuum at 60 °C overnight.

### 2.2. Materials Characterization

X-ray diffraction (XRD) patterns were acquired by using a Rigaku Smartlab diffractometer (Tokyo, Japan) with Cu-Kα radiation (λ = 1.5406 Å). X-ray photoelectron spectra (XPS) were conducted on a Thermo Fisher Scientific K-Alpha instrument (Waltham, MA, USA). Nitrogen adsorption–desorption isotherms were acquired at 77 K with a BSD-660M instrument (Beijing, China). The morphology of the samples was examined by field-emission scanning electron microscopy (FESEM; Hitachi-SU8010, Tokyo, Japan).

### 2.3. Electrochemical Measurements

All the electrochemical measurements were performed on a CHI 760E workstation at room temperature. In a typical three-electrode cell, the NiF_2_/NF was directly used as the working electrode, and the saturated Ag/AgCl electrode and platinum foil were used as the reference and counter electrode, respectively. Linear sweep voltammetry (LSV) curves were obtained at a scan rate of 5 mV s^−1^ with an iR compensation of 95%. The measured potentials were calibrated to the reversible hydrogen electrode (RHE) by the following equation: E_RHE_ = E_Ag/AgCl_ + 0.197 V + 0.059 × pH. Electrochemical impedance spectroscopy (EIS) measurements were conducted at an overpotential of 100 mV in the frequency range of 100 kHz~0.01 Hz with a 5 mV amplitude. Cyclic voltammetry (CV) measurements were carried out at increasing scan rates from 10 to 100 mV s^−1^ to estimate the double-layer capacitance (C_dl_). The electrochemically active surface area (ECSA) was evaluated based on the equation ECSA = C_dl_/C_s_, C_s_ = 0.06 mF cm^−2^ [34,35].

## 3. Results and Discussion

### 3.1. Structural Analysis

**Figure 1:**
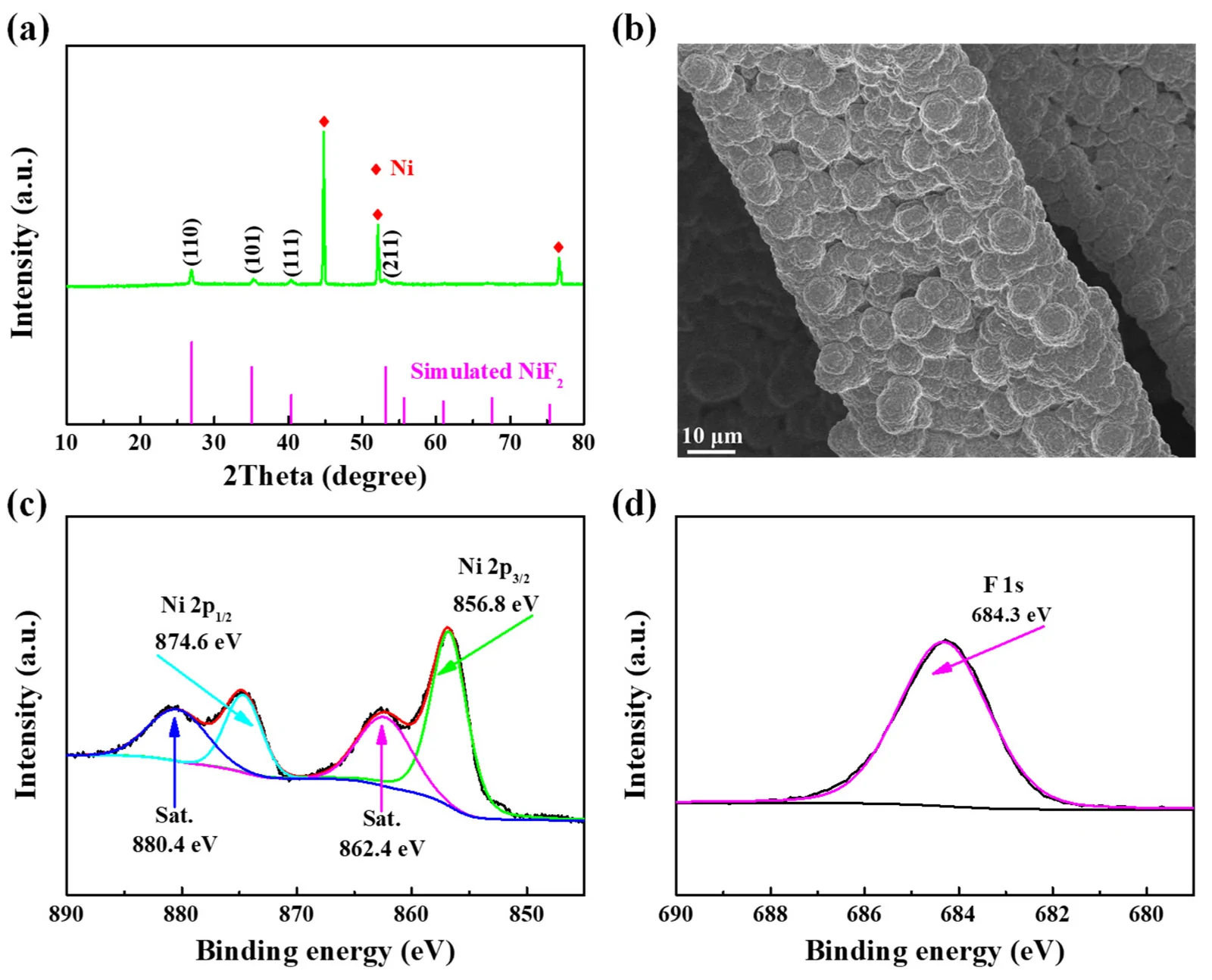
(**a**) XRD pattern, (**b**) SEM image, (**c**) Ni 2p and (**d**) F 1s spectra of NiF_2_/NF.

The in situ growth of NiF_2_ on nickel foam (NF) was first confirmed by XRD measurement. As illustrated in Figure 1a, the diffraction peaks of as-prepared fluoride emerged at 2θ = 26.9°, 35.2°, 40.4° and 53.2°, which were assigned to the (110), (101), (111) and (211) lattice facets of tetragonal NiF_2_. The other three peaks derived from the NF substrate could also be observed at 2θ = 44.7°, 52.1° and 76.7°. From Figure 1b, it can be seen that NiF_2_ grown on NF presented a spherical morphology with a rough surface. Furthermore, the mapping images of NiF_2_/NF demonstrated the uniform distribution of Ni and F elements (Figure S1). The specific surface area of NiF_2_/NF derived from the N_2_ adsorption–desorption isotherms was calculated to be 2.81 m^2^ g^−1^, with the pore size distribution mainly in the range of 2–5 nm (Figure S2), suggesting the presence of a mesopore structure. This porous configuration coupled with the conductive NF substrate is favorable to facilitate electrolyte penetration and mass transport, as well as to improve the accessibility of electroactive sites, thus resulting in enhanced HER performance [36,37]. XPS measurement was then conducted to investigate the chemical composition and valence of the as-prepared NiF_2_/NF sample. As shown in Figure 1c, the peaks of Ni 2p_1/2_ and Ni 2p_3/2_ for NiF_2_/NF were identified at the binding energy of 874.6 and 856.8 eV, respectively, with two satellite peaks at 880.4 and 862.4 eV, which were highly consistent with those of Ni^2+^ [35,38,39]. Additionally, the main peak of the high-resolution F 1s spectrum (Figure 1d) at 684.3 eV could be assigned to the metal-F bond [31,40,41], further indicating the successful preparation of NiF_2_.

### 3.2. Electrochemical Characterization

**Figure 2:**
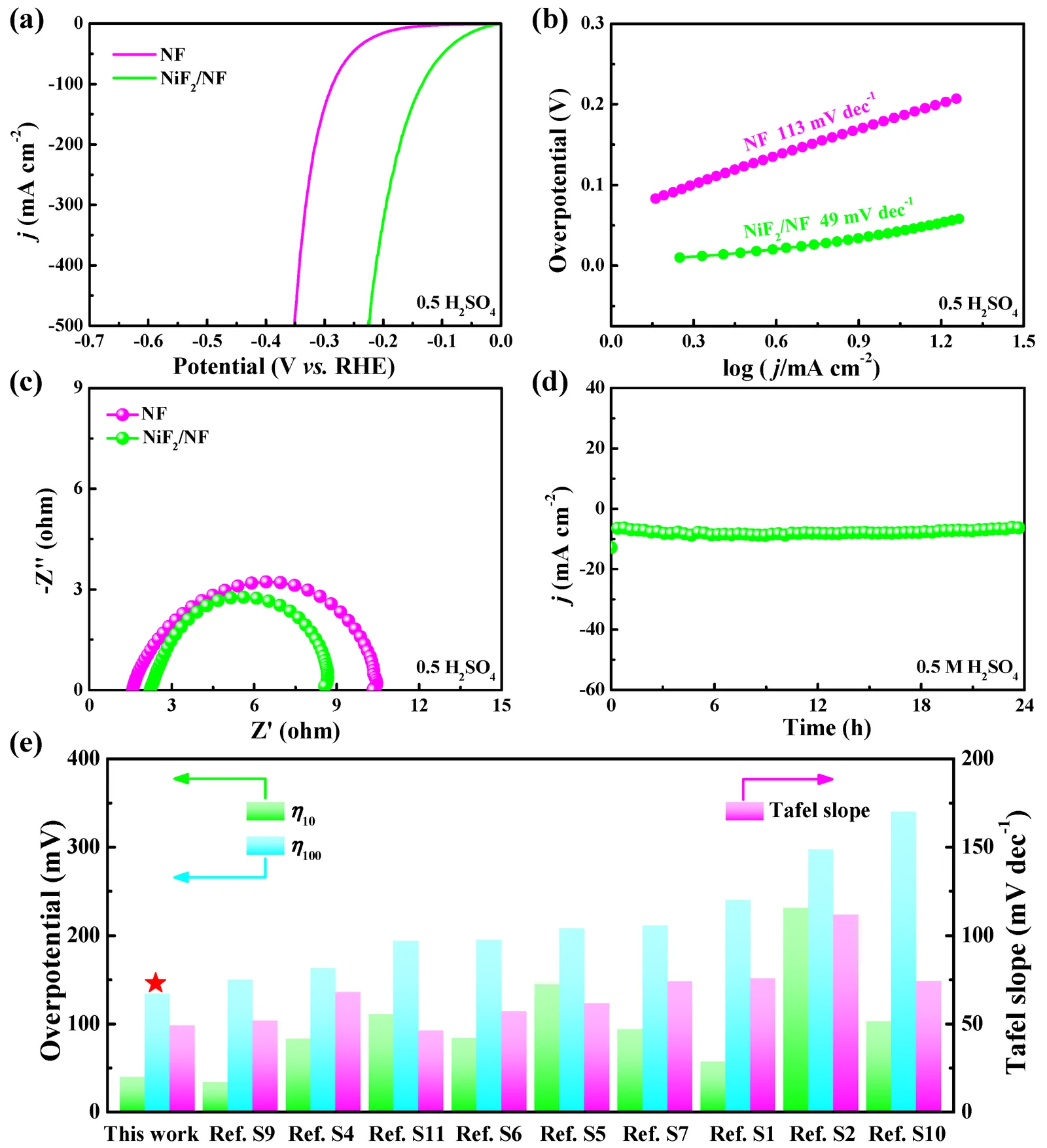
Electrochemical properties for HER in 0.5 M H_2_SO_4_. (**a**) LSV curves, (**b**) Tafel slopes and (**c**) Nyquist plots of NiF_2_/NF and NF. (**d**) Chronoamperometry curve of NiF_2_/NF for 24 h. (**e**) Comparison of electrocatalytic performance of NiF_2_/NF with other advanced electrocatalysts.

The electrocatalytic properties of NiF_2_/NF for the HER were first evaluated in 0.5 M H_2_SO_4_ solution by using a typical three-electrode cell. As seen in Figure 2a, the bare NF exhibited poor HER performance with an overpotential of 181 mV to achieve a current density of 10 mA cm^−2^. In sharp contrast, the self-supporting NiF_2_ microspheres showed excellent electrocatalytic activity for the HER, with ultralow overpotentials of only 40, 134, and 195 mV at current densities of 10, 100, and 300 mA cm^−2^, respectively. Even at the high current density of 500 mA cm^−2^, the overpotential of NiF_2_/NF was still as low as 226 mV, demonstrating its bright prospect for practical hydrogen production. In Figure 2b, the Tafel slope of NiF_2_/NF (49 mV dec^−1^) was much smaller than that of bare NF (113 mV dec^−1^), which was indicative of the superior electrocatalytic kinetics toward the HER. The outstanding HER performance of the NiF_2_/NF electrocatalyst was further confirmed by electrochemical impedance spectroscopy measurements (Figure 2c). It can be seen that NiF_2_/NF exhibited a smaller charge transfer resistance (*R*_ct_) than bare NF, implying faster electron transport kinetics [40,41,42]. The efficient HER activity of NiF_2_/NF can be attributed to an intrinsically faster reaction pathway under acidic conditions, in which abundant H^+^ can be directly reduced to adsorbed H* through the Volmer step (H^+^ + e^−^ → H*). Moreover, the in situ growth of NiF_2_ microspheres on conductive NF can facilitate charge transfer and ensure good electrode−electrolyte contact, thus leading to a superior reaction rate and HER activity.

Long-term stability is of great significance for the practical application of electrocatalysts and is also evaluated by chronoamperometry measurement. As expected, NiF_2_/NF presented good electrocatalytic durability in acidic media with a negligible current density decay after continuous operation for 24 h (Figure 2d). The electrocatalytic performance of NiF_2_/NF and other typical Ni-based electrocatalysts for the HER in previous studies are compared in Figure 2e and summarized in Table S1. It is found that the NiF_2_/NF in this current work exhibits much improved electrocatalytic activity, especially at high current density.

**Figure 3:**
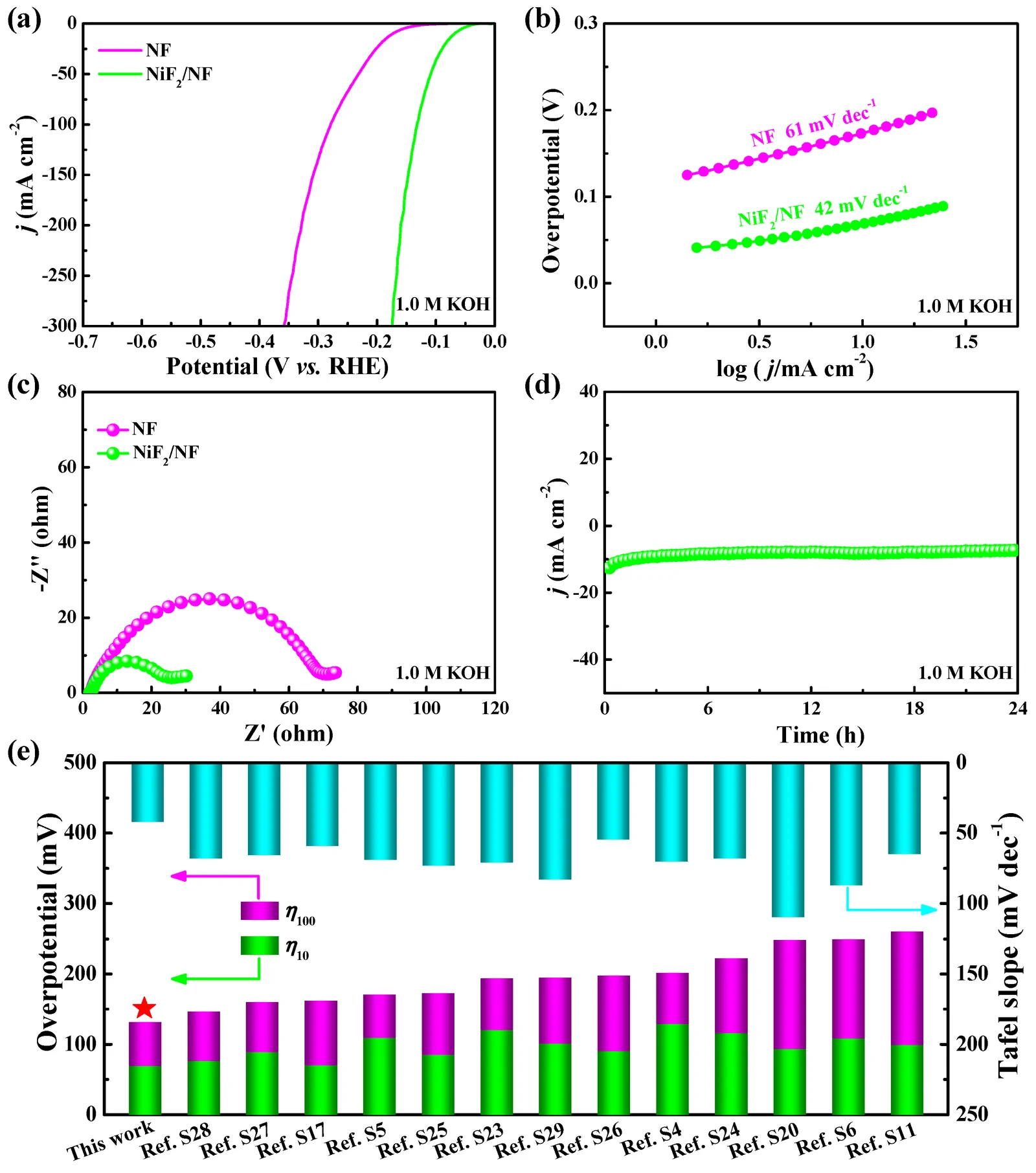
Electrochemical properties for HER in 1.0 M KOH. (**a**) LSV curves, (**b**) Tafel slopes and (**c**) Nyquist plots of NiF_2_/NF and NF. (**d**) Chronoamperometry curve of NiF_2_/NF for 24 h. (**e**) Comparison of electrocatalytic performance of NiF_2_/NF with other advanced electrocatalysts.

As depicted in Figure 3a, the NiF_2_/NF electrode also exhibited superior electrocatalytic HER activity in 1.0 M KOH solution with fairly low overpotentials of only 69, 132, and 175 mV to reach current densities of 10, 100, and 300 mA cm^−2^, respectively. Figure 3b shows the corresponding Tafel slopes of 42 and 61 mV dec^−1^ for NiF_2_/NF and bare NF, which indicates the accelerated reaction kinetics of NiF_2_/NF for the HER with the Volmer–Heyrovsky mechanism [43,44,45]. As illustrated in Figure 3c, the much smaller *R*_ct_ of NiF_2_/NF is favorable to facilitate electron migration and thus to promote HER activity. The double-layer capacitance (C_dl_), generally used to evaluate the electrochemical surface area (ECSA), was further investigated by CV measurement at increasing scan rates from 10 to 100 mV s^−1^ (Figure S3a,b). The NiF_2_/NF electrocatalyst can be seen to have a higher C_dl_ value of 6.7 mF cm^−2^ (Figure S3c), which is approximately double that of bare NF, demonstrating more active sites in NiF_2_/NF for efficient HER [42,44,46,47]. To reveal the intrinsic electrocatalytic activity of NiF_2_/NF, the current density of HER polarization curves was normalized by the ECSA (Figure S3d). Impressively, NiF_2_/NF can be seen to exhibit high intrinsic activity for the HER, with a large current density of 1.38 mA cm^−2^_ECSA_ at an overpotential of 175 mV. The above results indicate that the superior electrochemical kinetics (low Tafel slope), fast charge transport (small *R*_ct_), and abundant reaction sites (large ECSA) contribute greatly to the excellent HER activity of NiF_2_/NF. Figure 3d displayed the electrocatalytic stability of NiF_2_/NF, where the current density exhibited very limited change after a 24 h chronoamperometry test, clearly revealing its good durability. More importantly, the phase structure and morphology of NiF_2_/NF could be maintained after HER testing in both acidic and alkaline electrolytes (Figures S4 and S5), which explained the outstanding HER stability. It is noted that the peak positions of Ni 2p and F 1s slightly shifted towards the lower binding energy after HER testing in 1.0 M KOH (Figure S6), which might be ascribed to the partial conversion of Ni-F to Ni-OH [31,48]. Additionally, the weaker peak intensity of F 1s could be associated with the F^−^ leaching during the alkaline HER process [31]. Compared with other advanced HER electrocatalysts reported in the literature, this NiF_2_/NF showed a smaller Tafel slope and lower overpotential in an alkaline solution (Figure 3e and Table S2).

**Figure 4:**
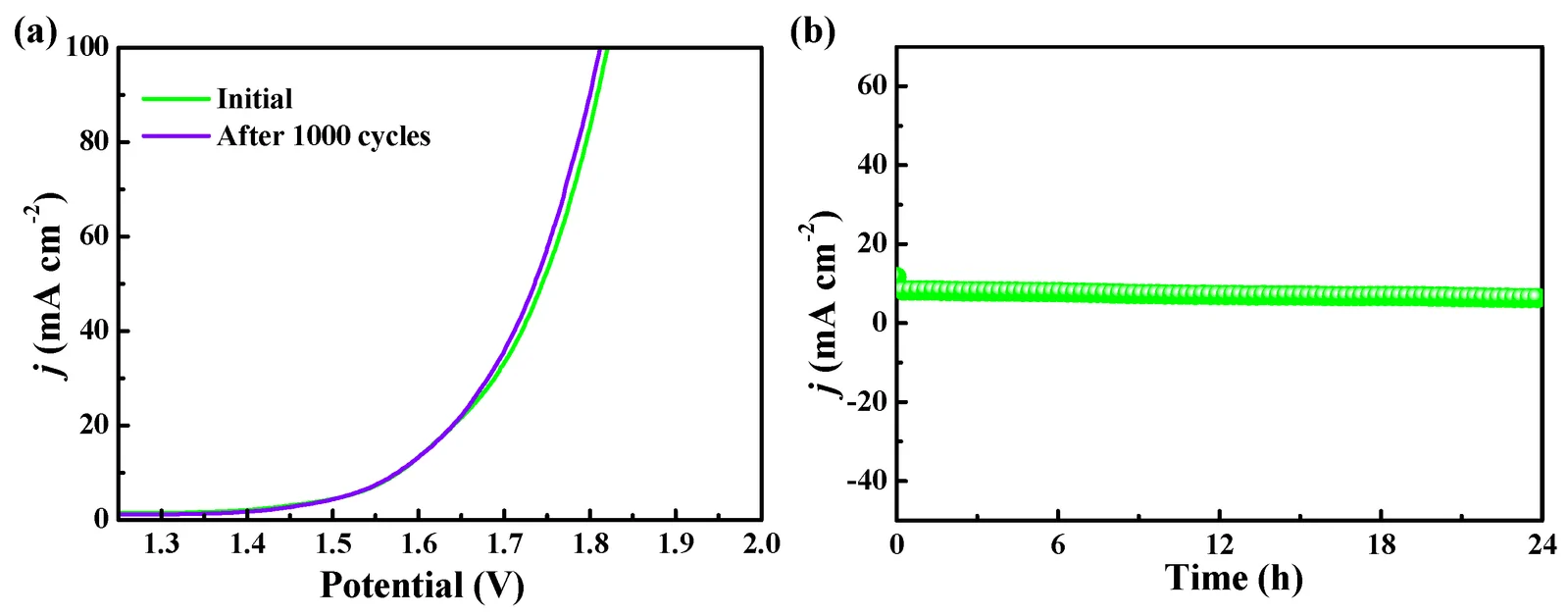
(**a**) LSV curves and (**b**) durability of NiF_2_/NF for water electrolysis in 1.0 M KOH.

Encouraged by its good HER and OER performance (Figure S7), the two-electrode electrolyzer assembled with the bifunctional NiF_2_/NF as both the cathode and anode was then employed to evaluate the capability for overall water splitting in 1.0 M KOH solution. Figure 4a plots the LSV curves of the as-prepared NiF_2_/NF electrode before and after 1000 cycles. It can be seen that NiF_2_/NF only required a low cell voltage of 1.57 V to drive the current density of 10 mA cm^−2^, demonstrating the superior electrocatalytic activity for water electrolysis. Furthermore, the chronoamperometry measurement with a negligible attenuation of current density also indicated its favorable electrocatalytic stability towards overall water splitting (Figure 4b). These results highlight that NiF_2_/NF can serve as an efficient electrocatalyst to split water into H_2_ and O_2_ under alkaline conditions.

## 4. Conclusions

In summary, a self-supporting NiF_2_/NF electrode has been prepared successfully through a facile one-step solvothermal approach. As electrocatalysts for HER, it can deliver current densities of 10, 100, and 300 mA cm^−2^ at low overpotentials of 40, 134, and 195 mV in 0.5 M H_2_SO_4_. Encouragingly, the overpotential was only 226 mV, even at a large current density of 500 mA cm^−2^. This NiF_2_/NF also exhibited favorable electrocatalytic HER activity in 1.0 M KOH, with low overpotentials of 69, 132, and 175 mV at 10, 100, and 300 mA cm^−2^, respectively. Furthermore, NiF_2_/NF can drive 10 mA cm^−2^ for overall water splitting at a low cell voltage of 1.57 V in an alkaline electrolyzer. This current work provided a facile strategy to design and construct efficient electrocatalysts for hydrogen production in both acidic and alkaline media.

## Data Availability

The original contributions presented in this study are included in the article and Supplementary Material. Further inquiries can be directed to the corresponding authors.

## Supplementary Materials

The following supporting information can be downloaded at https://www.mdpi.com/article/10.3390/ma19183871/s1: Figure S1: (a,b) Elemental mapping images and (c) EDX spectrum of NiF_2_/NF; Figure S2: Nitrogen adsorption–desorption isotherms of NiF_2_/NF. Inset is the corresponding pore size distribution plot; Figure S3: CV curves of (a) NiF_2_/NF and (b) NF at different scan rates towards HER in 1.0 M KOH. (c) The double-layer capacitance and (d) ECSA-normalized LSV curves of NiF_2_/NF and NF; Figure S4: XRD patterns of NiF_2_/NF after HER test in 0.5 M H_2_SO_4_ and 1.0 M KOH; Figure S5: SEM images of NiF_2_/NF after HER test in (a) 0.5 M H_2_SO_4_ and (b) 1.0 M KOH; Figure S6: (a) Ni 2p and (b) F 1s spectra of NiF_2_/NF after HER test in 0.5 M H_2_SO_4_ and 1.0 M KOH; Figure S7: LSV curves of NiF_2_/NF and NF towards OER with 80% iR compensation in 1.0 M KOH; Table S1: HER performance comparison of as-prepared NiF_2_/NF with other advanced electrocatalysts in 0.5 M H_2_SO_4_; Table S2: HER performance comparison of as-prepared NiF_2_/NF with other advanced electrocatalysts in 1.0 M KOH. Refs [49,50,51,52,53,54,55,56,57,58,59,60,61,62,63,64,65,66,67,68,69,70,71,72,73,74] are included in Supplementary Materials.
