# Supplementary material for: NiF2 Microspheres Grown on Nickel Foam for Efficient Hydrogen Evolution in Both Acidic and Alkaline Media
Source: Materials (Basel). 2026 Sep 11;19(18):3871. doi: 10.3390/ma19183871 (PMC13608445; doi:10.3390/ma19183871)
Supplement: Supplementary file 1 [file materials-19-03871-s001.zip › materials-4504435-supplementary.pdf]

NiF_2 Microspheres Grown on Nickel Foam for Efficient Hydrogen Evolution in Both Acidic and Alkaline Media

Jun Yang [1,*], Zenghui Wan [2], Long Yu [2] and Changlin Yu [1,*]

[1] School of Chemical Engineering, Guangdong University of Petrochemical Technology, Maoming 525000, China

[2] Guangdong Provincial Key Laboratory of Green and Intelligent Agricultural Production, School of Environmental Science and Engineering, Guangdong University of Petrochemical Technology, Maoming 525000, China

* Correspondence: yangjun@gdupt.edu.cn; yuchanglinjx@163.com

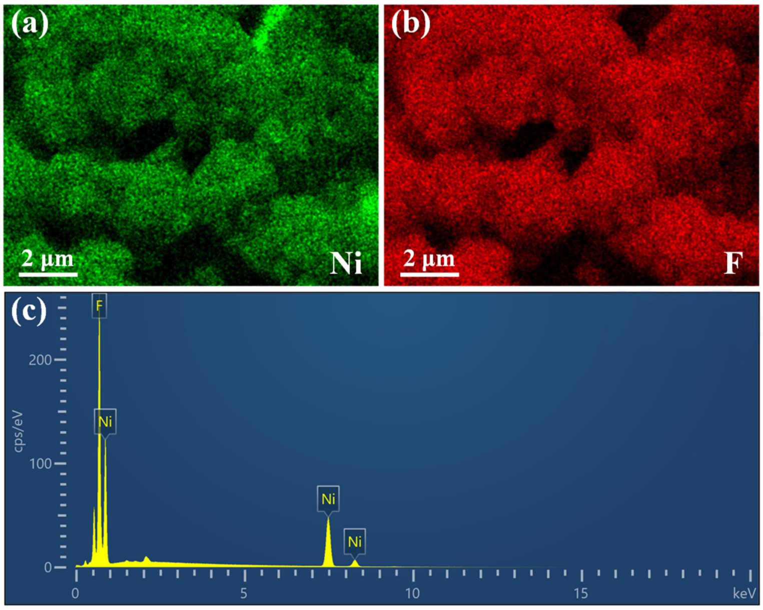

Figure S1. (a, b) Elemental mapping images and (c) EDX spectrum of NiF_2/NF.

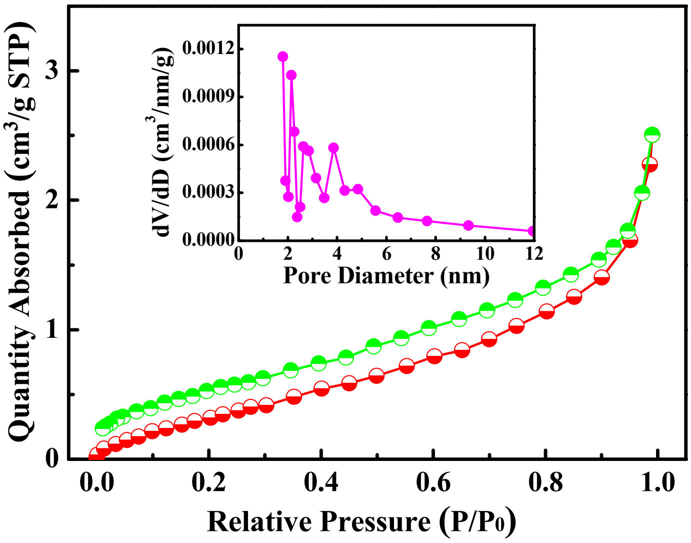

Figure S2. Nitrogen adsorption–desorption isotherms of NiF_2/NF. Inset is the corresponding pore size distribution plot.

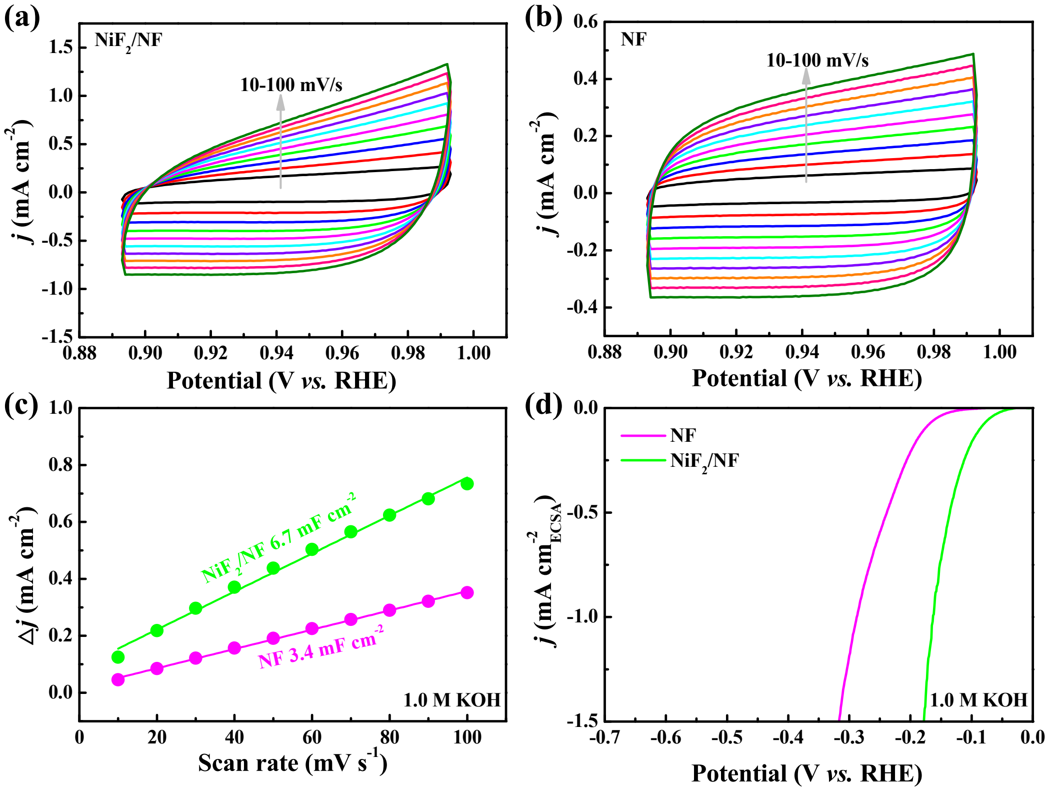

Figure S3. CV curves of (a) NiF_2/NF and (b) NF at different scan rates towards HER in 1.0 M KOH. (c) The double-layer capacitance and (d) ECSA-normalized LSV curves of NiF_2/NF and NF.

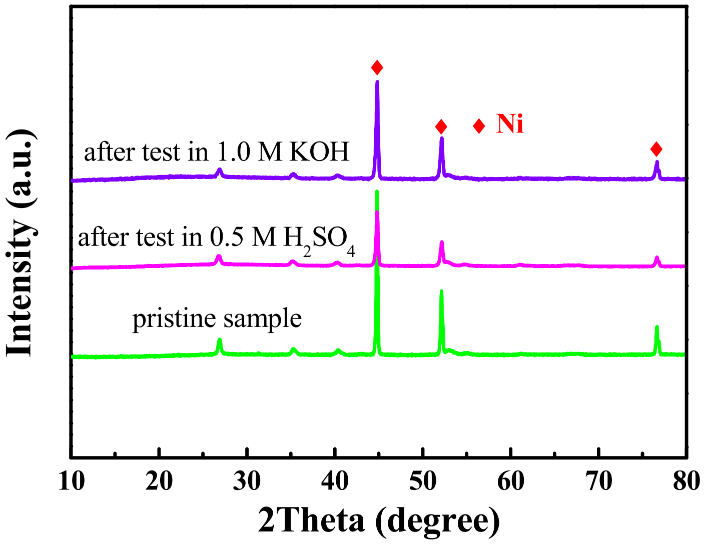

Figure S4. XRD patterns of NiF_2/NF after HER test in 0.5 M H_2SO_4 and 1.0 M KOH.

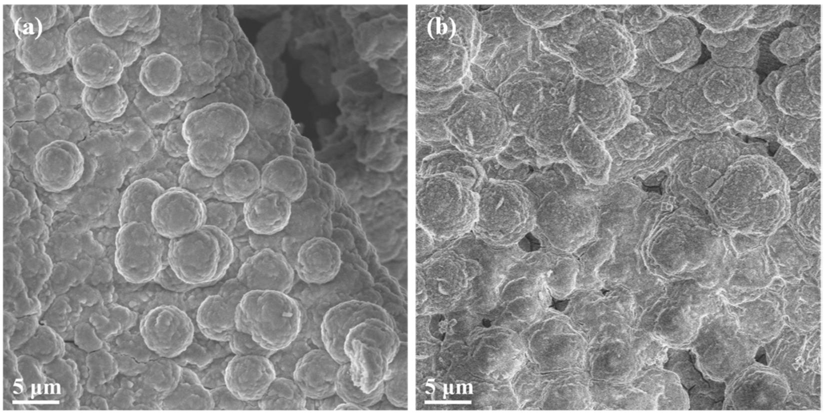

Figure S5. SEM images of NiF_2/NF after HER test in (a) 0.5 M H_2SO_4 and (b) 1.0 M KOH.

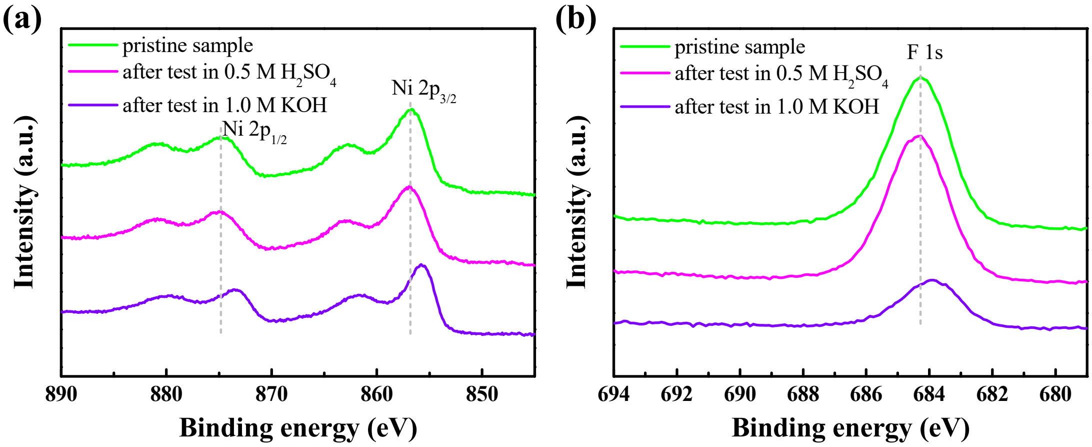

Figure S6. (a) Ni 2p and (b) F 1s spectra of NiF_2/NF after HER test in 0.5 M H_2SO_4 and 1.0 M KOH.

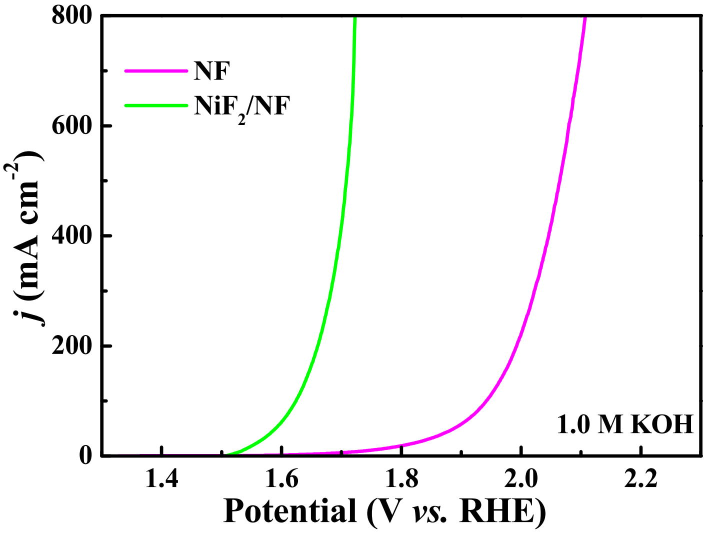

Figure S7. LSV curves of NiF_2/NF and NF towards OER with 80% iR compensation in 1.0 M KOH.

Table S1. HER performance comparison of as-prepared NiF_2/NF with other advanced electrocatalysts in 0.5 M H_2SO_4.

Electrocatalysts	η_{10} (mV)	η_{100} (mV)	Tafel slope (mV dec^{-1})	Ref.
C@$Cd_{0.9}Ni_{0.1}S$/CP	57	240	75.8	[1]
Co-Ni@CC	231	297	111.7	[2]
Ni/N/O-rGO-1/Ni	229	442	162	[3]
Ni-P/Ni/NF	83	163	68	[4]
Ni_2P(S)/NF	145	208	61.8	[5]
CoMnP/Ni_2P/NF	84	195	57	[6]
WS_2/Ni_5P_4-Ni_2P	94	211	74	[7]
Mn-NiCoP/NF	37	/	57.4	[8]
NiCoP@NC NA/NF	34	150	51.8	[9]
Ni_3S_2@NiSe/NF	103	340	74.2	[10]
Ni_3S_2-MoS_2/NF	111	194	46.2	[11]
N-MoS_x/Ni_3S_2-4@NF	51	/	47	[12]
Ni_4N/Ni_3N/NiO/CFs	75	/	48.5	[13]
NiF_2/NF	40	134	49	This work

Table S2. HER performance comparison of as-prepared NiF_2/NF with other advanced electrocatalysts in 1.0 M KOH.

Electrocatalysts	η_{10} (mV)	η_{100} (mV)	Tafel slope (mV dec^{-1})	Ref.
Ni-P/Ni/NF	129	202	70	[4]
Ni_2P(S)/NF	109	171	68.9	[5]
CoMnP/Ni_2P/NF	108	249	87	[6]
Mn-NiCoP/NF	67	/	67.5	[8]
NiCoP@NC NA/NF	37	186	53.9	[9]
Ni_3S_2-MoS_2/NF	99	260	65	[11]
Ni_4N/Ni_3N/NiO/CFs	250	/	72.6	[13]
F_{10}-Ni_3N	78	/	117	[14]
OH-Ni/Ni_3C	72	/	106.3	[15]
Ni@NiO-600	172	/	67.62	[16]
MoO_3/Ni-NiO	70	162	59	[17]
Ni_3S_2-FeS@NF	71	/	54.5	[18]
Fe/W-Ni_3S_2/NF	174	/	102	[19]
W-$FeNi_2S_4$/Ni_3S_2/NF	93	248	109.7	[20]
Ni_3Se_4-Ni_3N/NF	60	/	51.1	[21]
Ni_3Se_4/$NiSe_2$-A	106	/	74	[22]
Ni-S-P/NF	120	194	71	[23]
Ni_2P/NF	116	222	68	[24]
Mo-NiP_x/NiS_y	85	173	73	[25]
NiS_2-Ni_2P/CC	90	198	54.6	[26]
NiP_2/$NiSe_2$	89	160	65.7	[27]
Ni_2P-$Ni_{12}P_5$/NF	76	147	68	[28]
Ni_2P/Co(OH)F-300@NF	101	195	83	[29]
NiF_3/Ni_2P@CC-2	121	/	75	[30]
R-CoF_2/CC	54	/	56	
R-NiF_2/CC	101	/	91	[31]
R-$FeF_3\cdot(H_2O)_{0.33}$/CC	159	/	95	
NiF_2/NF	69	132	42	This work

References

1. Lee, J.; Shin, H.; Jeong, H.; Jang, D.; Kim, Y.; Im, Y.; Kang, M. Autonomous interface stabilization via Ni doping, sulfur vacancy regulation, and carbon encapsulation for durable hydrogen evolution in acidic media. *Small* **2025**, *21*, 2504031.
2. Yu, H.; Li, J.; Yang, N.; Zhao, D.; Yang, B.; Hou, Y.; Xie, G. Ultrafast Joule heating method for synthesizing CoNi nanoalloy catalysts for electrocatalytic hydrogen evolution. *J. Alloys Compd.* **2025**, *1019*, 179101.
3. Hou, Z.; Jiang, H.; Guo, Y.; Huang, K.; Zhao, F.; Xu, Y.; Peng, P.; Zou, S.; Yan, J.; Zhang, J. Enhancing acidic hydrogen evolution through pyrrolic nitrogen-doped reduced graphene oxide triggering two-electron oxygen reduction. *Inorg. Chem. Front.* **2024**, *11*, 4318–4328.
4. Zhang, J.T.; Zhang, Z.; Ji, Y.F.; Yang, J.D.; Fan, K.; Ma, X.Z.; Wang, C.; Shu, R.Y.; Chen, Y. Surface engineering induced hierarchical porous $Ni_{12}P_5$-Ni_2P polymorphs catalyst for efficient wide pH hydrogen production. *Appl. Catal. B* **2021**, *282*, 119609.
5. Xu, J.; Li, T.T.; Zhang, L.; He, B.; Feng, D.L.; Zhou, Y. One-step synthesis of anionic S-substitution toward $Ni_2P(S)$ nanowires on nickel foam for enhanced hydrogen evolution reaction. *Int. J. Energy Res.* **2021**, *45*, 16973–16983.
6. Liu, M.Z.; Sun, Z.; Li, S.Y.; Nie, X.W.; Liu, Y.F.; Wang, E.D.; Zhao, Z.K. Hierarchical superhydrophilic/superaerophobic CoMnP/Ni_2P nanosheet-based microplate arrays for enhanced overall water splitting. *J. Mater. Chem. A* **2021**, *9*, 22129–22139.
7. Yu, S.H.; Chen, W.Z.; Wang, H.Y.; Pan, H.; Chua, D.H.C. Highly stable tungsten disulfide supported on a self-standing nickel phosphide foam as a hybrid electrocatalyst for efficient electrolytic hydrogen evolution. *Nano Energy* **2019**, *55*, 193–202.
8. Yu, X.; Xu, S.R.; Wang, Z.; Cheng, X.H.; Du, Y.S.; Chen, G.; Sun, X.P.; Wu, Q. An Mn-doped NiCoP flower-like structure as a highly efficient electrocatalyst for hydrogen evolution reaction in acidic and alkaline solutions with long duration. *Nanoscale* **2021**, *13*, 11069–11076.
9. Cao, B.; Cheng, Y.; Hu, M.H.; Jing, P.; Ma, Z.X.; Liu, B.C.; Gao, R.; Zhang, J. Efficient and durable 3D self-supported nitrogen-doped carbon-coupled nickel/cobalt phosphide electrodes: Stoichiometric ratio regulated phase- and morphology-dependent overall water splitting performance. *Adv. Funct. Mater.* **2019**, *29*, 1906316.
10. Shaikh, N.; Mukhopadhyay, I.; Ray, A. Heterointerfaces of nickel sulphides and selenides on Ni-foam as efficient bifunctional electrocatalysts in acidic environments. *J. Mater. Chem. A* **2022**, *10*, 12733–12746.
11. Gu, X.D.; Zheng, S.J.; Huang, X.B.; Yuan, H.F.; Li, J.P.; Kundu, M.; Wang, X.G. Hybrid Ni_3S_2-MoS_2 nanowire arrays as a pH-universal catalyst for accelerating the hydrogen evolution reaction. *Chem. Commun.* **2020**, *56*, 2471–2474.
12. Ma, X.; Chen, W.R.; Li, Q.; Xue, L.J.; Peng, C. Nitrogen-doped hierarchical heterostructured aerophobic MoS_x/Ni_3S_2 nanowires by one-pot synthesis: System engineering and synergistic effect in electrocatalysis of hydrogen evolution reaction. *Energy Environ. Mater.* **2021**, *4*, 658–663.
13. Liu, Q.; Fu, T.; Xiao, H.; Yu, Y.; Che, Z.; Zhang, Y.; Chen, A.; Li, M.; Liu, T. Constructing the Ni_4N/Ni_3N heterointerface of the bicontinuous Ni_4N/Ni_3N/NiO/CFs catalyst with pore connectivity effects for electrocatalytic hydrogen generation. *Carbon Neutralization* **2025**, *4*, e70024.
14. Qin, H.; Li, J.; Lin, G.; Yuan, K.; Yang, H.; Ye, Y.; Jin, T.; Cheng, F.; Jiao, L. Tuning surface coordination environment of Ni_3N by fluorine modification for efficient methanol electrooxidation assisted hydrogen evolution. *Adv. Mater.* **2025**, *37*, 2507573.
15. Wen, Q.; Duan, J.; Wang, W.; Huang, D.; Liu, Y.; Shi, Y.; Fang, J.; Nie, A.; Li, H.; Zhai, T. Engineering a local free water enriched microenvironment for surpassing platinum hydrogen evolution activity. *Angew. Chem. Int. Ed.* **2022**, *61*, e202206077.

16. Li, R.; Pu, Z.; Zhou, R.; Li, Y.; Huang, Y.; Li, S.; Yang, J.; Zhang, D.; Pi, M. In situ controllable construction of Ni@NiO Schottky heterojunctions for electrocatalytic hydrogen evolution. *J. Mater. Chem. C* **2024**, *12*, 18849–18855.
17. Li, X.P.; Wang, Y.; Wang, J.J.; Da, Y.M.; Zhang, J.F.; Li, L.L.; Zhong, C.; Deng, Y.D.; Han, X.P.; Hu, W.B. Sequential electrodeposition of bifunctional catalytically active structures in MoO_3/Ni-NiO composite electrocatalysts for selective hydrogen and oxygen evolution. *Adv. Mater.* **2020**, *32*, 2003414.
18. Liu, W.; Wang, F.; Wang, A.; Guo, Y.; Yin, H.; Wang, D. Boosting alkaline hydrogen evolution reaction by modulating D-band center in bimetallic sulfide Ni_3S_2-FeS heterointerfaces. *Small* **2025**, *21*, 2411028.
19. Wang, S.; Yuan, D.; Sun, S.; Huang, S.; Wu, Y.; Zhang, L.; Dou, S.X.; Liu, H.K.; Dou, Y.; Xu, J. Iron, tungsten dual-doped nickel sulfide as efficient bifunctional catalyst for overall water splitting. *Small* **2024**, *20*, 2311770.
20. Jiang, J.; Su, H.; Song, S.; Liu, W.; Li, N.; Gao, Y.; Ge, L. W-doped $FeNi_2S_4$/Ni_3S_2/NF with interfacial effect as efficient bifunctional electrocatalyst for overall water splitting. *Nano Res.* **2023**, *16*, 12116–12125.
21. Alemayehu, D.D.; Tsai, M.-C.; Tsai, M.-H.; Yang, C.-C.; Chang, C.-C.; Chang, C.-Y.; Moges, E.A.; Lakshmanan, K.; Nikodimos, Y.; Su, W.-N.; et al. Heterogeneous interfaces of Ni_3Se_4 nanoclusters decorated on a Ni_3N surface enhance efficient and durable hydrogen evolution reactions in alkaline electrolyte. *J. Am. Chem. Soc* **2025**, *147*, 16047–16059.
22. Guo, K.; Wang, Y.; Huang, J.; Li, H.; Peng, Y.; Xu, C. Symbiotic Ni_3Se_4/Ni heterostructure induced by unstable $NiSe_2$ for enhanced hydrogen generation. *Chem. Eng. J.* **2023**, *454*, 140488.
23. Xu, Q.L.; Gao, W.L.; Wang, M.; Yuan, G.; Ren, X.N.; Zhao, R.; Zhao, S.D.; Wang, Q.F. Electrodeposition of NiS/Ni_2P nanoparticles embedded in amorphous $Ni(OH)_2$ nanosheets as an efficient and durable dual-functional electrocatalyst for overall water splitting. *Int. J. Hydrogen Energy* **2020**, *45*, 2546–2556.
24. Wu, Y.T.; Wang, H.; Ji, S.; Pollet, B.G.; Wang, X.Y.; Wang, R.F. Engineered porous Ni_2P-nanoparticle/Ni_2P-nanosheet arrays via the Kirkendall effect and Ostwald ripening towards efficient overall water splitting. *Nano Res.* **2020**, *13*, 2098–2105.
25. Wang, J.; Zhang, M.; Yang, G.; Song, W.; Zhong, W.; Wang, X.; Wang, M.; Sun, T.; Tang, Y. Heterogeneous bimetallic Mo-NiP_x/NiS_y as a highly efficient electrocatalyst for robust overall water splitting. *Adv. Funct. Mater.* **2021**, *31*, 2101532.
26. Wang, Y.; Yin, R.; Yuan, L.; Guo, X.; Zheng, X.; Fan, Q.; Duan, Z.; Liu, Y.; Zhang, J.; Xiong, S. Uniform sub-5 nm crystalline nickel-based heterojunctions for overall water splitting electrocatalysis. *ACS Energy Lett.* **2025**, *10*, 837–844.
27. Yang, L.; Huang, L.T.; Yao, Y.H.; Jiao, L.F. In-situ construction of lattice-matching NiP_2/$NiSe_2$ heterointerfaces with electron redistribution for boosting overall water splitting. *Appl. Catal. B* **2021**, *282*, 119584.
28. Wang, Z.K.; Wang, S.Y.; Ma, L.X.; Guo, Y.J.; Sun, J.; Zhang, N.; Jiang, R.B. Water-induced formation of Ni_2P-$Ni_{12}P_5$ interfaces with superior electrocatalytic activity toward hydrogen evolution reaction. *Small* **2021**, *17*, 2006770.
29. Guo, D.; Duan, P.; Yan, Z.; An, Y.; Hu, Y.; Yao, W.; Dou, Y. Enhanced hydrogen evolution performance of Ni_2P/Co(OH)F@NF heterostructures via low-temperature phosphorization. *Int. J. Hydrogen Energy* **2026**, *227*, 154601.
30. Wang, K.L.; Huang, W.; Cao, Q.H.; Zhao, Y.J.; Sun, X.J.; Ding, R.; Lin, W.W.; Liu, E.H.; Gao, P. Engineering NiF_3/Ni_2P heterojunction as efficient electrocatalysts for urea oxidation and splitting. *Chem. Eng. J.* **2022**, *427*, 130865.

31. Ji, P.X.; Yu, R.H.; Wang, P.Y.; Pan, X.L.; Jin, H.H.; Zheng, D.Y.; Chen, D.; Zhu, J.W.; Pu, Z.H.; Wu, J.S.; et al. Ultra-fast and in-depth reconstruction of transition metal fluorides in electrocatalytic hydrogen evolution processes. *Adv. Sci.* **2022**, *9*, 2103567.
